# Transposition of cardiovascular outcome trial effects to the real-world population of patients with type 2 diabetes

**DOI:** 10.1186/s12933-021-01300-y

**Published:** 2021-05-10

**Authors:** V. Sciannameo, P. Berchialla, A. Avogaro, G. P. Fadini, DARWIN-T2D Network, Agostino Consoli, Gloria Formoso, Giovanni Grossi, Achiropita Pucci, Giorgio Sesti, Francesco Andreozzi, Giuseppe Capobianco, Adriano Gatti, Riccardo Bonadonna, Ivana Zavaroni, Alessandra DeiCas, Giuseppe Felace, Patrizia Li Volsi, Raffaella Buzzetti, Gaetano Leto, Gian Pio Sorice, Paola D’Angelo, Susanna Morano, Antonio Carlo Bossi, Edoardo Duratorre, Ivano Franzetti, Paola Silvia Morpurgo, Emanuela Orsi, Fabrizio Querci, Massimo Boemi, Federica D’Angelo, Massimiliano Petrelli, Gianluca Aimaretti, Ioannis Karamouzis, Franco Cavalot, Giuseppe Saglietti, Giuliana Cazzetta, Silvestre Cervone, Eleonora Devangelio, Olga Lamacchia, Salvatore Arena, Antonino Di Benedetto, Lucia Frittitta, Carla Giordano, Salvatore Piro, Manfredi Rizzo, Roberta Chianetta, Carlo Mannina, Roberto Anichini, Giuseppe Penno, Anna Solini, Bruno Fattor, Enzo Bonora, Massimo Cigolini, Annunziata Lapolla, Nino Cristiano Chilelli, Natalino Simioni, Vera Frison, Carmela Vinci

**Affiliations:** 1 Unit of Biostatistics, Epidemiology and Public Health, Department of Cardiac, Thoracic, Vascular Sciences and Public Health, University of Padova, Padua, Italy; 2 Department of Clinical and Biological Sciences, University of Turin, Turin, Italy; 3 Department of Medicine, University of Padova, Via Giustiniani 2, 35128 Padova, Italy

## Abstract

**Background:**

Transferring results obtained in cardiovascular outcome trials (CVOTs) to the real-world setting is challenging. We herein transposed CVOT results to the population of patients with type 2 diabetes (T2D) seen in routine clinical practice and who may receive the medications tested in CVOTs.

**Methods:**

We implemented the post-stratification approach based on aggregate data of CVOTs and individual data of a target population of diabetic outpatients. We used stratum-specific estimates available from CVOTs to calculate expected effect size for the target population by weighting the average of the stratum-specific treatment effects according to proportions of a given characteristic in the target population. Data are presented as hazard ratio (HR) and 95% confidence intervals.

**Results:**

Compared to the target population (n = 139,708), the CVOT population (n = 95,816) was younger and had a two to threefold greater prevalence of cardiovascular disease. EMPA-REG was the CVOT with the largest variety of details on stratum-specific effects, followed by TECOS, whereas DECLARE and PIONEER-6 had more limited stratum-specific information. The post-stratification HR estimate for 3 point major adverse cardiovascular event (MACE) based on EMPA-REG was 0.88 (0.74–1.03) in the target population, compared to 0.86 (0.74–0.99) in the trial. The HR estimate based on LEADER was 0.88 (0.77–0.99) in the target population compared to 0.87 (0.78–0.97) in the trial. Consistent results were obtained for SUSTAIN-6, EXSCEL, PIONEER-6 and DECLARE. The effect of DPP-4 inhibitors observed in CVOTs remained neutral in the target population.

**Conclusions:**

Based on CVOT stratum-specific effects, cardiovascular protective actions of glucose lowering medications tested in CVOTs are transferrable to a much different real-world population of patients with T2D.

**Supplementary Information:**

The online version contains supplementary material available at 10.1186/s12933-021-01300-y.

## Background

Prevention of cardiovascular disease is a major objective of diabetes care [1]. In the past decade, cardiovascular outcome trials (CVOTs) have been performed in patients with type 2 diabetes (T2D) with the primary aim of demonstrating safety of glucose lowering medications (GLMs) concerning the risk of cardiovascular events [2, 3]. Some of these CVOTs were designed to test superiority and some eventually found lower rates of cardiovascular events among patients randomized to active GLM compared to those randomized to placebo plus standard care [4]. This is the case of CVOTs investigating glucagon-like peptide-1 receptor agonists (GLP-1RA) and sodium glucose cotransporter-2 inhibitors (SGLT2i) [5–7]. Yet, in order to rapidly collect the target number of events to demonstrate safety, CVOTs enrolled patients with high or very-high cardiovascular risk at baseline, such as those with prior cardiovascular events or a history of established cardiovascular disease [4]. More recently, CVOTs included subgroups of patients with multiple cardiovascular risk factors but without established cardiovascular disease [8–10]. Nonetheless, the overall cardiovascular risk of patients enrolled in most CVOTs was much higher than in the population of patients with T2D who could receive the new GLM in routine clinical practice. Other remarkable differences have been highlighted between CVOT populations and the typical outpatients with T2D, including age and sex distribution [11]. Indeed, small proportions of patients with T2D would satisfy enrolment criteria of CVOTs on GLP-1RA or SGLT2i and even smaller proportions actually have CVOT-like characteristics [12, 13]. For these reasons, there has been an intense debate on whether results of CVOTs can be transferred to the general real-world population of patients with T2D, irrespective of their cardiovascular risk profile [14, 15]. This is a clinically-relevant question informing on which and how many patients would benefit from GLM with CVOT-proven cardiovascular protective effect in routine care. Though prior studies have addressed the generalizability of trial populations, no study so far has explored whether CVOT findings, i.e. the drug’s effect on the outcome(s), can be transferred to a target population with different characteristics from that of the trial.

We herein used an innovative approach to transpose the effects of GLP-1RA or SGLT2i observed in the respective CVOTs to a large unselected target population of patients who were followed under routine specialist care and could potentially be prescribed such medications.

## Methods

### Selection of CVOTs

Since the method for transposing trial effects to the target population relies on the availability of stratum-specific information of effect [16], we selected CVOTs reporting the hazard ratio for the primary outcome in various subgroups of patients based on clinical characteristics of the trial population. CVOTs were identified based on literature search and then selected based on whether key information were available. The search string was: (“cardiovascular” AND “outcome” AND “randomized” AND “trial” AND “type 2 diabetes”). As the primary outcome of interest, we elected the 3-point major adverse cardiovascular events (3P-MACE), a composite of non-fatal myocardial infarction, non-fatal stroke, or cardiovascular death. Eventual co-primary outcome(s) were also considered [10]. For comparison, we also performed the same analysis on typical CVOTs that have shown neutral effects of the drugs under investigation with respect to the rate of cardiovascular events, such as those performed with dipeptidyl peptidse-4 inhibitors (DPP-4i).

### Target population

In Italy, GLP-1RA and SGLT2i can be prescribed only by diabetes specialists. Therefore, as a target population of individuals with T2D who could receive such medications in real-life, we used the DARWIN-T2D (data from the real-world in type 2 diabetes) database [17]. DARWIN-T2D was a retrospective multicentre study collecting data from 46 diabetes specialist outpatient clinics in Italy. While DARWIN-T2D included longitudinal assessment of patients initiating a few selected glucose lowering medications [18], it also recorded cross-sectional data on all patients with T2D at their last available visit each participating Centre [19]. All patients aged 18 years or older and with a diagnosis of T2D were included, yielding a population of about 281,000 patients, evaluated between 2015 and 2016. Therefore, this was an unselected population of adults with T2D attending diabetes clinics, which is estimated to represent about 20% of the entire population of individuals with T2D attending diabetes clinics in Italy [20, 21]. Study design and methods for data collection, including definition of variables, have been described previously in detail [17]. Briefly, we collected data on demographics, anthropometrics, risk factors, laboratory values, complications, and medications at the last available visit up to December 31st, 2016. The study was conducted according to the principles of the Declaration of Helsinki and approved by ethics committees at all participating centres. Based on national regulations on retrospective studies with anonymous data, patients’ informed consent was waived.

### Transposition and statistical analysis

**Fig. 1:**
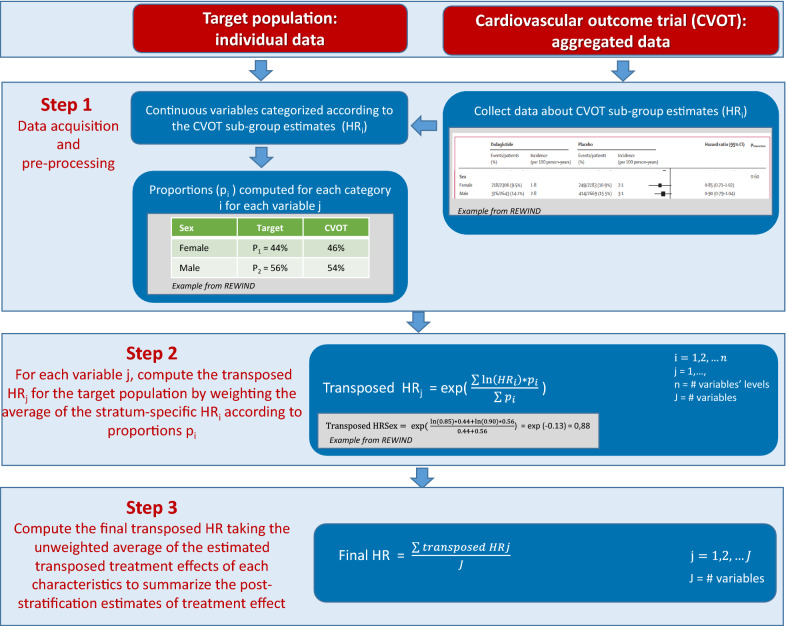
A flow-chart of the transposition method. The figure illustrates the three-step procedure used to transpose a cardiovascular outcome trial (CVOT) result to the target population. An example from the REWIND study is described in the text

The method for transposition is escribed as a flow-chart in Fig. 1. The most diffused setting in which transposition of trial effect is performed is when individual-level data for both the trial and the target population are available. In this case, patients in the trials are weighted by their probability to meet the inclusion criteria. Then, an outcome analysis is performed within the weighted trial data [22]. Contrariwise, in our study we disposed of individual-level data for the target population and aggregated data for CVOTs. As a consequence, we could not perform outcome analyses (i.e. weighting using simulated individual data or weighting using the method of moments, which require individual-level data for the trial), but we could use stratum-specific trial estimates to transpose the trial effect to the target population [23]. More in detail, we implemented a post-stratification approach based on aggregate data of CVOTs and individual data of the target population in DARWIN-T2D, with an inverse approach compared to that described previously [16, 23]. We retained only patients from the target populations for whom all variables were available for each specific trial transposition. We excluded patients with missing data because no method has been validated to pool results of the transposition approach from multiple imputed datasets. For each CVOT transposition, we have used definitions of cardiovascular disease based on the real-world data closer to those of the CVOT, with some adjustments done as previously described [12]. Heart failure was defined using ICD-9 codes reported in the DARWIN-T2D database, which may differ from the definition used in CVOTs [24]. Continuous variables in DARWIN-T2D were categorized according to the CVOT stratum-specific estimates. Then, the subgroup-specific estimates of treatment effect in the CVOT and proportions of the categorized characteristics in DARWIN-T2D were used to calculate the treatment effect for the target population by weighting the average of the stratum-specific treatment effects according to proportions of a given characteristic in the target population.

As a simple example, let us consider the “gender” variable in the REWIND trial, where 46% of participants were women. In the target population, 44% of subjects were women. In REWIND, the stratum-specific HR estimates were 0.85 (95% C.I. 0.71–1.02) for females and 0.90 (95% C.I. 0.79–1.04) for males. Then, the weighted HR estimate for the variable “gender” is computed by the formula:

$${\text{Transposed HR}} = {\text{exp }}\left( {\frac{{\sum \ln \left( {HR_{i} } \right)*p_{i} }}{{\sum p_{i} }}} \right) ,i = 1.2$$

where $p_{i}$ states for proportion in DARWIN-T2D in the level $i$ of the variable. In our example, the transposed HR was obtained as $\frac{{{\text{ln}}\left( {0.85} \right){*}0.44 + {\text{ln}}\left( {0.90} \right){*}0.56}}{0.44 + 0.56} = - 0.{13}$, that exponentiated leads to a HR = 0.88 (Additional file 1: Table S1).

The standard deviations across strata were pooled to obtain the 95% confidence interval. The calculation was performed for one characteristic at a time. Then, the unweighted average of the estimated transposed treatment effect of each characteristic was used to summarize the post-stratification estimates of treatment effect. Analyses were performed using R version 3.5.2 [25].

## Results

The analysis was conducted only for CVOTs having usable data for transposition. For example, transposition could not be performed for the CANVAS study because reporting of stratum-specific effects was not accompanied by numbers of patients in each stratum [26]. The analysis was not performed for HARMONY [27] because albiglutide has never become clinically available. Only the primary endpoint was considered, because stratum-specific effects were most of the times not available for individual components of the 3P-MACE and other secondary outcomes. For the DECLARE study, transposition was performed for both co-primary outcomes [10].

The number of variables that defined stratum-specific effects ranged from a maximum of 28 for EMPA-REG [28] to a minimum of 6 for DECLARE [10]. Therefore, as shown in Table 1, different number and types of variables were used to perform transposition for different CVOT. This indicates that results of the transposition analysis cannot be compared across CVOTs.

**Table 1:** Post-stratification variables

	EMPA-REG	TECOS	SAVOR-TIMI	SUSTAIN-6	LEADER	EXSCEL	REWIND	PIONEER-6	DECLARE
Duration of diabetes		X	X	X	X	X	X		X
Age	X	X	X	X	X	X	X	X	X
Sex	X	X	X	X	X	X	X	X	
HbA1c	X	X	X	X	X	X	X	X	
BMI	X	X	X	X	X	X	X	X	
Body weight			X						
Systolic blood pressure	X	X							
Diastolic blood pressure	X	X							
Established CVD	X			X	X	X	X	X	
Prior MI or Stroke	X			X			X	X	
PAD	X								
Previous MI	X								X
Heart failure		X	X	X	X	X			X
CVD risk factors	X			X	X			X	X
Only cerebrovascular disease	X								
eGFR	X	X		X	X	X		X	X
Urinary albumin/creatinine ratio	X		X						
Anti-diabetic therapy					X				
Insulin	X	X	X	X		X			
Metfromin	X	X	X						
Sulphonylurea	X	X	X						
Thiazolidinediones	X	X	X						
DPP-4i	X					X			
Anti-hypertive therapy	X		X						
RAS blockers		X	X						
Calcium channel blockers	X	X							
Beta blockers	X	X							
Diuretics	X	X	X						
Aspirin	X								
Statin	X	X	X						
Europe	X	X	X	X	X	X	X		
Ethnicity	X			X	X				
White	X	X	X	X	X	X	X	X	
Number of variables	28	20	18	14	13	12	9	9	6

For each cardiovascular outcome trial, we report which variables were used for post-stratification transposition to the target population

*BMI* body mass index, *CVD* cardiovascular disease, *PAD* peripheral arterial disease, *MI* myocardial infarction, *eGFR* estimated glomerular filtration rate, *DPP-4* dipeptidyl peptidase-4, *RAS* renin angiotensin system

After excluding patients with missing data of key variables, the target population of the DARWIN-T2D study was composed of a total of 139,726 patients, but not all information was available for all patients. Table 2 shows clinical characteristics of patients in the target population compared to those of patients enrolled in CVOTs (n = 95,816). The CVOT population was younger, with a shorter diabetes duration, was more often obese, and had a two to threefold greater prevalence of cardiovascular disease, reflected by more frequent use of cardiovascular medications. Yet, median albumin excretion rate was lower than in the target population, likely because patients with advanced renal disease were excluded from CVOTs. Among glucose lowering medications, patients enrolled in CVOTs had more frequent use of sulphonylurea and insulin. On average, only 41.9% patients enrolled in the selected trials were recruited in Europe and 75.0% were white. The substantial difference between the CVOT and the target population was expected and forms the rationale for performing the transposition analysis.

**Table 2:** Clinical characteristics

Variable	Target population		CVOTs	
	Number	Value	Number	Value
Duration of diabetes, years	139,700	12.1 (9.4)	71,636	11.7
Age, years	139,708	68.8 (11.2)	95,816	64.4
Sex male, %	139,726	57.1	95,816	66.4
HbA1c, % mmol/mol	132,717	7.3 (1.3) 56 (9)	95,816	8.0 64
BMI, kg/m^2^	126,994	29.6 (5.5)	95,816	31.6
Body weight, kg	128,431	80.8 (17.1)	39,332	89.1
Systolic blood pressure, mm Hg	104,305	137.2 (18.4)	64,572	135.6
Diastolic blood pressure, mm Hg	104,226	77.5 (9.5)	47,412	77.3
Established CVD, %	139,726	28.9	95,816	67.5
PAD, %	139,726	6.0	53,603	14.4
Previous MI, %	97,074	11.7	50,820	38.8
Heart failure, %	139,726	1.4	92,633	13.5
eGFR, ml/min/1.73 m^2^	113,593	75.7 (24.5)	83,179	76.7
Albumin creatinine ratio, mg/g (median)	113,775	22.6	41,064	1.4
Glucose-lowering therapy, %	139,726	93.3	95,816	95.0
Insulin, %	130,380	33.5	95,816	39.5
Metformin, %	130,380	71.3	95,816	77.3
Sulphonylurea, %	130,380	27.5	95,816	42.5
Thiazolidinediones, %	130,380	5.0	78,656	4.1
DPP-4 inhibitors, %	130,080	23.3	95,816	16.3
SGLT-2 inhibitors, %	130,080	4.4	95,816	13.8
GLP-1 receptor agonists, %	130,080	5.1	95,816	21.1
Anti-hypertensive therapy, %	117,632	80.1	37,592	92.3
RAS blockers, %	117,632	67.0	92,633	79.2
Calcium channel blockers, %	117,632	25.1	49,080	32.8
Beta blockers, %	117,632	31.5	92,633	56.7
Diuretics, %	117,632	19.2	52,263	41.9
Statin, %	117,632	61.1	95,816	75.3
Aspirin, %	117,632	50.6	95,816	68.3

Data are presented as mean (SD) for continuous variables or as percentage for categorical variables. The number of patients with available information for each variable is shown for both populations

**Fig. 2:**
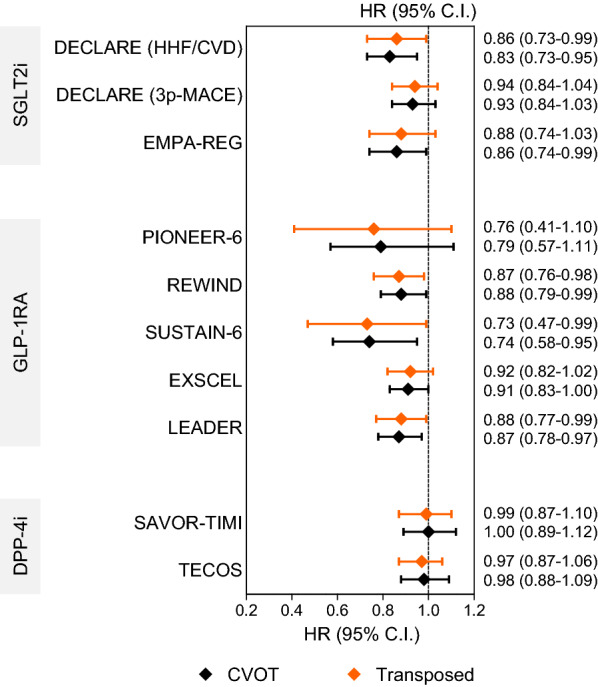
Comparison between observed and transposed effects. The forest plot reports hazard ratios and 95% confidence intervals (C.I.) for 3-point major adverse cardiovascular events (3P-MACE) and the second co-primary endpoint in DECLARE in the original cardiovascular outcome trial (CVOTs, black) and after transposition to the target population (red). *HHF* hospitalization for heat failure, *CVD* cardiovascular death

After transposition to the target population, the estimated HR was significantly lower than 1.0 for LEADER [29], SUSTAIN-6 [30], REWIND [9] and DECLARE [10] (co-primary endpoint of cardiovascular death or hospitalization for heart failure). The HR for 3P-MACE in patients randomized to empagliflozin in EMPA-REG was 0.86 (95% C.I. 0.74–0.99) and changed to 0.88 (95% C.I. 0.74–1.03) when transposed to the target population. Figure 2 compares the HR (95% C.I.) of the effect observed in CVOT with the corresponding HR (95% C.I.) obtained after transposition to the target population. For each CVOT, subgroup-weighted mean of stratum-specific estimates from CVOTs are given in Additional file 1: Table S1–S10.

The effect on 3p-MACE observed in EXSCEL [31], PIONEER-6 [32] and DECLARE [10] was not significant in the trial and remained so after transposition. As expected, the transposed estimate of DPP-4i effects using stratum-specific data from TECOS [33] or SAVOR-TIMI [34] yielded neutral results also in the target population.

## Discussion

Despite major differences between patients with T2D enrolled in CVOTs and patients with T2D seen in routine clinical practice [12–14], our transposition analysis shows that most significant results of CVOTs apply to the target real world population.

Due to the specific population of patients enrolled in CVOTs, doubts have been cast on whether significant protective effects observed for some of the active drugs being tested could be transferred to the entire population of patients who could receive such medications in clinical practice [12]. In most CVOTs, subgroup analyses performed for most, but not all, stratification variables showed no substantial heterogeneity in the effect, claiming for a direct clinical transferability of the findings. Nonetheless, several trends of interaction and a few nominally significant interactions between the assigned treatment and stratification variables may yield overall significant effects when transposed to a much different target population.

To the best of our knowledge, there is no prior attempt to transpose effects of diabetes medications observed in CVOTs to a real-world population, with quantitative estimates. Thus, our new findings can help reducing inertia in the use of GLM for which solid cardiorenal protective data exist [35].

The post-stratification method described by Hong et al. can be used to generalize trial results without accessing individual-level data [16]. The gold standard approach would require individual data from both CVOTs and the target population to predict probabilities of being sampled in the trial and to reweight trial participants to reflect the target population of patient characteristics. However, accessing individual data of multiple CVOTs sponsored by different companies can be harnessed by compliance issues and conflicts of interest. Alternative methods, as the one we used, are subjected to biases and based on some critical assumptions. Specifically, this approach requires only categorical variables and is effective only when a small number of variables are taken into account [22]. In addition, it can be used only for one variable at a time, making the assumption of no correlation between them, which does not necessarily hold true. Further, results of transposition is strictly dependent on which and how many strata are available for the trial, such that the effects of important variables not considered for stratification are disregarded. Moreover, this approach is generally used when individual data are available for CVOT and aggregate data are available for the target population [16]. In our case, we applied the method with individual data for the target population and aggregate data for the CVOTs. Another limitation is the need to know the proportion of the target population in each strata [16] and the proportion of missing data in DARWIN-T2D could lead to biased results.

It is important to note that most CVOTs reporting superiority of active drugs versus placebo for cardiovascular protection was confirmed after transposition to the real-world population. This is the case for LEADER, SUSTAIN-6, REWIND, and DECLARE (second co-primary endpoint), while it was not for EMPA-REG Outcome. The reasons why the significantly lower rate of 3P-MACE among patients randomized to empagliflozin in the EMPA-REG Outcome trial was not significant after transposition to the target population can be manifold. These include the presence of nominally significant heterogeneity observed in subgroups of patients divided by age and baseline HbA1c [28], the 2:1 ratio between patients on empagliflozin and those on placebo yielding small numbers of patients in some strata, and the large number of variables (n = 28) that composed strata used for transposition. With regards to the latter point, when we transposed EMPA-REG Outcome with the 6 strata used to transpose DECLARE, we obtained an estimated HR of 0.85 (95% C.I. 0.70–0.99) for the target population. To gather further insight into this point, we repeated the analysis to evaluate which stratifications made the HR transposed from EMPA-REG Outcome not significant, by backward elimination ordered by standard deviation of the estimate (from larger to smaller): the HR was still significant (0.85; 95% C.I. 0.71–0.99) with 11 of the initial 28 strata. However, this approach has not been validated. Therefore, to rule out that this finding was biased by assumptions of the method used, transposition for EMPA-REG should be repeated using individual-level patients’ data. In any case, the simple fact that fully transposed HR for EMPA-REG had an upper limit crossing unity does not imply that EMPA-REG results are less generalizable to the target population than other CVOT’s, because the observed and transposed HR were quite similar.

It is important to note that only 30% to 50% of patients enrolled in CVOTs were recruited in Europe, questioning generalizability of the findings to European populations. We also would like to note that the target population addressed in this study might not be representative of patients with T2D in other countries. We included only patients followed at specialist outpatient clinics because, in Italy, only diabetes specialists but not general practitioners, can prescribe GLP-1RA, SGLT2i and DPP-4i [20]. Therefore, further transferability of our findings to the general population of patients with T2D, including those not attending diabetes clinics, needs confirmation. Finally, we transposed the CVOT drug’s effect as if all patients of the target population could and would receive that drug. We did not apply CVOT inclusion/exclusion criteria because we aimed to estimate the effect in an unselected target population. However, not all real-world patients with T2D are candidate for a therapy with GLP-1RA or SGLT2i, because of possible contraindications (e.g. advanced kidney disease) and eventual regulatory restrictions. With regards to the latter, we argue that, if significant benefits of GLP-1RA and SGLT2i on unselected patients with T2D hold true, regulatory limitations might be relieved in order to improve access to the best available care.

## Conclusions

Notwithstanding the above-described limitations, we herein provide the first estimate that cardiovascular protection by diabetes drugs investigated in CVOTs could apply to a very different and highly heterogeneous population of patients with T2D seen in routine care.

## Supplementary Information

**Additional file 1:** **Table S1.** REWIND. **Table S2.** SUSTAIN-6. **Table S3.** DECLARE HHF/CVD. **Table S4.** DECLARE MACE. **Table S5.** EMPA-REG. **Table S6.** LEADER. **Table S7.** PIONEER-6. **Table S8.** TECOS. **Table S9.** SAVOR-TIMI. **Table S10.** EXCEL.

## Data Availability

Data used for this study are either publicly available or available from the corresponding author at a reasonable request.

## Abbreviations

3P-MACE: 3-Point major adverse cardiovascular events
CANVAS: Canagliflozin Cardiovascular Assessment Study
CI: Confidence interval
CVOTs: Cardiovascular outcome trials
DARWIN-T2D: Data from the real-world in type 2 diabetes
DECLARE: Dapagliflozin effect on cardiovascular events
DPP-4i: Dipeptidyl peptidse-4 inhibitors
EMPA-REG: Empagliflozin remove excess glucose
EXSCEL: Exenatide Study of Cardiovascular Event Lowering Trial
GLM: Glucose lowering medications
GLP-1RA: Glucagon-like peptide-1 receptor agonists
HR: Hazard ratio
LEADER: Liraglutide Effect and Action in Diabetes: Evaluation of Cardiovascular Outcome Results
PIONEER-6: Peptide Innovation for Early Diabetes Treatment 6
SGLT2i: Sodium glucose co-transporter-2 inhibitors
SUSTAIN-6: Trial to Evaluate Cardiovascular and Other Long-term Outcomes with Semaglutide
T2D: Type 2 diabetes
