# Supplementary material for: Transposition of cardiovascular outcome trial effects to the real-world population of patients with type 2 diabetes
Source: Cardiovasc Diabetol. 2021 May 10;20:103. doi: 10.1186/s12933-021-01300-y (PMC8112047; doi:10.1186/s12933-021-01300-y)
Supplement: Supplementary file 1 — Additional file 1: Table S1. REWIND. Table S2. SUSTAIN-6. Table S3. DECLARE HHF/CVD. Table S4. DECLARE MACE. Table S5. EMPA-REG. Table S6. LEADER. Table S7. PIONEER-6. Table S8. TECOS. Table S9. SAVOR-TIMI. Table S10. EXCEL. [file 12933_2021_1300_MOESM1_ESM.docx]

Additional file 1

**Table S1**. **REWIND**. CVOT cardiovascular outcome trial, CVD cardiovascular disease, BMI Body Mass Index, MI myocardial infarction, HR hazard ratio, low CI and high CI refer to the low and high 95% confidence interval (CI) limit, respectively.

| **Characteristic*s*** | **CVOT estimates** | | | **Proportions** | | **Sub-group weighted mean of strato-specific CVOT estimates** | | | | |
| --- | --- | --- | --- | --- | --- | --- | --- | --- | --- | --- |
|  | *HR* | *low CI* | *high CI* | *Darwin* | *CVOT* | *HR* | *low CI* | | *high CI* | |
| Age < 66 yrs | 0.92 | 0.78 | 1.09 | 0.34 | 0.53 | 0.88 | | 0.77 | | 0.99 |
| Age ≥ 66 yrs | 0.86 | 0.74 | 1.00 | 0.65 | 0.47 |  | |  | |  |
| Sex: Female | 0.85 | 0.71 | 1.02 | 0.44 | 0.46 | 0.88 | 0.76 | | 0.99 | |
| Sex: Male | 0.90 | 0.79 | 1.04 | 0.56 | 0.54 |  |  | |  | |
| Duration diabetes < 5 yrs | 0.84 | 0.66 | 1.06 | 0.27 | 0.24 | 0.88 | 0.77 | | 0.99 | |
| Duration diabetes in [5,10] yrs | 0.89 | 0.73 | 1.09 | 0.22 | 0.30 |  |  | |  | |
| Duration diabetes > 10 yrs | 0.90 | 0.77 | 1.06 | 0.51 | 0.46 |  |  | |  | |
| CVD: Yes | 0.87 | 0.74 | 1.02 | 0.20 | 0.31 | 0.87 | 0.75 | | 0.99 | |
| CVD: No | 0.87 | 0.74 | 1.02 | 0.80 | 0.63 |  |  | |  | |
| Hba1c < 7.2 % | 0.90 | 0.76 | 1.06 | 0.29 | 0.47 | 0.87 | 0.76 | | 0.98 | |
| Hba1c ≥ 7.2 % | 0.86 | 0.74 | 1.00 | 0.65 | 0.53 |  |  | |  | |
| BMI < 32 kg/m^2^ | 0.94 | 0.81 | 1.09 | 0.34 | 0.54 | 0.90 | 0.80 | | 1.01 | |
| BMI ≥ 32 kg/m^2^ | 0.82 | 0.69 | 0.96 | 0.14 | 0.46 |  |  | |  | |
| Region: Europe | 0.77 | 0.65 | 0.90 | 1.00 | 0.44 | 0.77 | 0.67 | | 0.87 | |
| MI or Stroke: Yes | 0.79 | 0.66 | 0.96 | 0.09 | 0.21 | 0.92 | 0.80 | | 1.04 | |
| MI or Stroke: No | 0.93 | 0.81 | 1.07 | 0.91 | 0.79 |  |  | |  | |
| Race: White | 0.90 | 0.79 | 1.02 | 1.00 | 0.76 | 0.90 | 0.79 | | 1.01 | |
| **CVOT estimate 0.88 (0.79-0.99)** | | | | | | **Transposed estimate 0.87 (0.76-0.98)** | | | | |

**Table S2. SUSTAIN-6**. CVOT cardiovascular outcome trial, CVD cardiovascular disease, BMI Body Mass Index, MI myocardial infarction, HR hazard ratio, low CI and high CI refer to the low and high 95% confidence interval (CI) limit, respectively.

| **Characteristic*s*** | **CVOT estimates** | | | **Proportions** | | **Sub-group weighted mean of strato-specific CVOT estimates** | | |
| --- | --- | --- | --- | --- | --- | --- | --- | --- |
|  | *HR* | *low CI* | *high CI* | *Darwin* | *CVOT* | *HR* | *low CI* | *high CI* |
| Sex: Female | 0.84 | 0.54 | 1.31 | 0.44 | 0.39 | 0.75 | 0.49 | 1.00 |
| Sex: Male | 0.68 | 0.50 | 0.92 | 0.56 | 0.61 |  |  |  |
| Age < 65 yrs | 0.74 | 0.52 | 1.05 | 0.32 | 0.39 | 0.73 | 0.49 | 0.96 |
| Age ≥ 65 yrs | 0.72 | 0.51 | 1.02 | 0.67 | 0.48 |  |  |  |
| BMI < 30 kg/m^2^ | 0.58 | 0.39 | 0.87 | 0.29 | 0.36 | 0.67 | 0.42 | 0.93 |
| BMI ≥ 30 kg/m^2^ | 0.84 | 0.61 | 1.16 | 0.20 | 0.64 |  |  |  |
| Hba1c ≤ 8.5 % | 0.72 | 0.50 | 1.03 | 0.45 | 0.56 | 0.72 | 0.47 | 0.97 |
| Hba1c > 8.5 % | 0.74 | 0.52 | 1.04 | 0.07 | 0.44 |  |  |  |
| Duration diabetes ≤ 10 yrs | 0.73 | 0.48 | 1.12 | 0.54 | 0.35 | 0.73 | 0.48 | 0.98 |
| Duration diabetes > 10 yrs | 0.73 | 0.54 | 0.99 | 0.47 | 0.65 |  |  |  |
| Egfr < 60 ml/min/1.73 m^2^ | 0.84 | 0.57 | 1.25 | 0.12 | 0.28 | 0.72 | 0.46 | 0.96 |
| Egfr ≥ 60 ml/min/1.73 m^2^ | 0.67 | 0.48 | 0.92 | 0.33 | 0.72 |  |  |  |
| Insulin: No | 0.52 | 0.33 | 0.81 | 0.31 | 0.42 | 0.65 | 0.38 | 0.93 |
| Insulin: Yes | 1.02 | 0.64 | 1.62 | 0.16 | 0.32 |  |  |  |
| CVD: Yes | 0.72 | 0.55 | 0.93 | 0.20 | 0.83 | 0.72 | 0.49 | 0.95 |
| CVD risk factors: Yes | 1.00 | 0.41 | 2.46 | 0.10 | 0.17 | 1.00 | 0.63 | 1.37 |
| Heart Failure: No | 0.64 | 0.48 | 0.86 | 0.98 | 0.83 | 0.64 | 0.39 | 0.90 |
| Heart Failure: Yes | 1.03 | 0.64 | 1.66 | 0.02 | 0.17 |  |  |  |
| MI or stroke = No | 0.70 | 0.47 | 1.04 | 0.91 | 0.59 | 0.70 | 0.44 | 0.97 |
| MI or stroke = Yes | 0.76 | 0.55 | 1.05 | 0.09 | 0.41 |  |  |  |
| Region; Europe | 0.62 | 0.34 | 1.13 | 1.00 | 0.19 | 0.62 | 0.36 | 0.88 |
| Race: White | 0.76 | 0.58 | 1.00 | 1.00 | 0.83 | 0.76 | 0.51 | 1.01 |
| Ethnicity: Not Hispanic or Latinos | 0.74 | 0.57 | 0.96 | 1.00 | 0.85 | 0.74 | 0.50 | 0.98 |
|  | **CVOT estimate: 0.74 (0.58-0.95)** | | | | | **Transposed estimate: 0.73 (0.47-0.99)** | | |

**Table S3. DECLARE HHF/CVD.** HHF hospitalization for heat failure, CVOT cardiovascular outcome trial, MI myocardial infarction, HR hazard ratio, low CI and high CI refer to the low and high 95% confidence interval (CI) limit, respectively.

| **Characteristic*s*** | **CVOT estimates** | | | **Proportions** | | **Sub-group weighted mean of strato-specific CVOT estimates** | | |
| --- | --- | --- | --- | --- | --- | --- | --- | --- |
|  | *HR* | *low CI* | *high CI* | *Darwin* | *CVOT* | *HR* | *low CI* | *high CI* |
| Heart Failure: Yes | 0.79 | 0.63 | 0.99 | 0.02 | 0.10 | 0.84 | 0.64 | 1.04 |
| Heart Failure: No | 0.84 | 0.72 | 0.99 | 0.98 | 0.90 |  |  |  |
| Age < 65 yrs | 0.88 | 0.72 | 1.07 | 0.32 | 0.54 | 0.86 | 0.69 | 1.04 |
| Age in [65; 75] yrs | 0.77 | 0.63 | 0.94 | 0.31 | 0.40 |  |  |  |
| Age ≥ 75 yrs | 0.94 | 0.65 | 1.36 | 0.36 | 0.06 |  |  |  |
| Egfr < 60 ml/min/1.73 m^2^ | 0.78 | 0.55 | 1.09 | 0.12 | 0.07 | 0.83 | 0.66 | 1.01 |
| Egfr in [60; 90) ml/min/1.73 m^2^ | 0.79 | 0.66 | 0.95 | 0.20 | 0.45 |  |  |  |
| Egfr ≥ 90 ml/min/1.73 m^2^ | 0.96 | 0.77 | 1.19 | 0.13 | 0.48 |  |  |  |
| Duration diabetes < 5 | 1.08 | 0.87 | 1.35 | 0.32 | 0.22 | 0.94 | 0.81 | 1.07 |
| Duration diabetes in [5;10) | 1.02 | 0.83 | 1.25 | 0.22 | 0.28 |  |  |  |
| Duration diabetes in [10; 15) | 0.94 | 0.77 | 1.15 | 0.17 | 0.23 |  |  |  |
| Duration diabetes in [15; 20) | 0.92 | 0.71 | 1.18 | 0.12 | 0.14 |  |  |  |
| Duration diabetes ≥20 yrs | 0.67 | 0.52 | 0.86 | 0.18 | 0.13 |  |  |  |
| Previous MI: No | 0.85 | 0.72 | 1.00 | 0.56 | 0.79 | 0.85 | 0.67 | 1.02 |
| Previous MI: Yes | 0.81 | 0.65 | 1.00 | 0.07 | 0.21 |  | | |
|  | **CVOT estimate: 0.83 (0.73-0.95)** | | | | | **Transposed estimate: 0.86 (0.73-0.99)** | | |

**Table S4. DECLARE MACE**. MACE 3-point major adverse cardiovascular events, CVOT cardiovascular outcome trial, MI myocardial infarction, HR hazard ratio, low CI and high CI refer to the low and high 95% confidence interval (CI) limit, respectively.

| **Characteristic*s*** | **CVOT estimates** | | | **Proportions** | | **Sub-group weighted mean of strato-specific CVOT estimates** | | |
| --- | --- | --- | --- | --- | --- | --- | --- | --- |
|  | *HR* | *low CI* | *high CI* | *Darwin* | *CVOT* | *HR* | *low CI* | *high CI* |
| Heart Failure: Yes | 1.01 | 0.81 | 1.27 | 0.02 | 0.10 | 0.92 | 0.80 | 1.05 |
| Hear Failure: No | 0.92 | 0.82 | 1.02 | 0.98 | 0.90 |  |  |  |
| Age < 65 yrs | 0.93 | 0.81 | 1.08 | 0.32 | 0.54 | 0.91 | 0.77 | 1.04 |
| Age in [65; 75) yrs | 0.97 | 0.83 | 1.13 | 0.31 | 0.40 |  |  |  |
| Age ≥ 75 yrs | 0.84 | 0.61 | 1.15 | 0.36 | 0.06 |  |  |  |
| Egfr < 60 ml/min/1.73 m^2^ | 0.92 | 0.69 | 1.23 | 0.12 | 0.07 | 0.94 | 0.81 | 1.07 |
| Egfr in [60; 90) ml/min/1.73 m^2^ | 0.95 | 0.82 | 1.09 | 0.20 | 0.45 |  |  |  |
| Egfr ≥ 90 ml/min/1.73 m^2^ | 0.94 | 0.80 | 1.10 | 0.13 | 0.48 |  |  |  |
| Duration diabetes < 5 yrs | 1.08 | 0.87 | 1.35 | 0.32 | 0.22 | 0.94 | 0.81 | 1.07 |
| Duration diabetes in [5;10) yrs | 1.02 | 0.83 | 1.25 | 0.22 | 0.28 |  |  |  |
| Duration diabetes in [10; 15) yrs | 0.94 | 0.77 | 1.15 | 0.17 | 0.23 |  |  |  |
| Duration diabetes in [15;20) yrs | 0.92 | 0.71 | 1.18 | 0.12 | 0.14 |  |  |  |
| Duration diabetes ≥ 20 yrs | 0.67 | 0.52 | 0.86 | 0.18 | 0.13 |  |  |  |
| Previous MI: No | 1.00 | 0.88 | 1.13 | 0.56 | 0.79 | 0.98 | 0.85 | 1.12 |
| Previous MI: Yes | 0.84 | 0.72 | 0.99 | 0.07 | 0.21 |  | | |
|  | **CVOT estimate : 0.93 (0.84-1.03)** | | | | | **Transposed estimate: 0.94 (0.84-1.04)** | | |

**Table S5. EMPA-REG**. CVOT cardiovascular outcome trial, CVD cardiovascular disease, BMI Body Mass Index, MI myocardial infarction, SBP systolic Blood Pressure, DBP Diastolic Blood Pressure, HR hazard ratio, low CI and high CI refer to the low and high 95% confidence interval (CI) limit, respectively.

| **Characteristic*s*** | **CVOT estimates** | | | **Proportions** | | **Sub-group weighted mean of strato-specific CVOT estimates** | | |
| --- | --- | --- | --- | --- | --- | --- | --- | --- |
|  | *HR* | *low CI* | *high CI* | *Darwin* | *CVOT* | *HR* | *low CI* | *high CI* |
| Age < 65 yrs | 1.04 | 0.84 | 1.29 | 0.32 | 0.55 | 0.80 | 0.68 | 0.93 |
| Age ≥ 65 yrs | 0.71 | 0.59 | 0.87 | 0.67 | 0.45 |  |  |  |
| Angiotensing converting: No | 0.77 | 0.56 | 1.07 | 0.14 | 0.19 | 0.84 | 0.71 | 0.97 |
| Angiotensing converting: Yes | 0.88 | 0.75 | 1.04 | 0.27 | 0.81 |  |  |  |
| Antihypertensive: No | 0.94 | 0.45 | 1.95 | 0.08 | 0.05 | 0.87 | 0.74 | 0.99 |
| Antihypertensive: Yes | 0.85 | 0.73 | 0.99 | 0.34 | 0.95 |  |  |  |
| Acetylsalicyclic acid: No | 0.80 | 0.57 | 1.12 | 0.22 | 0.17 | 0.83 | 0.71 | 0.95 |
| Acetylsalicyclic acid: Yes | 0.87 | 0.74 | 1.02 | 0.20 | 0.83 |  |  |  |
| Beta blockers: No | 0.90 | 0.70 | 1.17 | 0.29 | 0.35 | 0.88 | 0.75 | 1.01 |
| Beta blockers: Yes | 0.83 | 0.70 | 1.00 | 0.13 | 0.65 |  |  |  |
| BMI < 30 kg/m^2^ | 0.74 | 0.60 | 0.91 | 0.28 | 0.48 | 0.83 | 0.71 | 0.96 |
| BMI ≥ 30 kg/m^2^ | 0.98 | 0.80 | 1.21 | 0.20 | 0.52 |  |  |  |
| Calcium channel blockers : No | 0.87 | 0.73 | 1.05 | 0.31 | 0.67 | 0.86 | 0.73 | 0.99 |
| Calcium channel blockers: Yes | 0.83 | 0.65 | 1.06 | 0.10 | 0.33 |  |  |  |
| Cerebrovascular disease | 1.15 | 0.74 | 1.78 | 0.02 | 0.14 | 1.15 | 1.01 | 1.29 |
| CVD risk factors | 0.79 | 0.61 | 1.04 | 0.10 | 0.19 | 0.79 | 0.69 | 0.89 |
| Diuretics: No | 0.83 | 0.67 | 1.02 | 0.34 | 0.57 | 0.84 | 0.72 | 0.96 |
| Diuretics: Yes | 0.88 | 0.71 | 1.07 | 0.08 | 0.43 |  |  |  |
| Dpp4: No | 0.81 | 0.70 | 0.95 | 0.36 | 0.89 | 0.90 | 0.77 | 1.03 |
| Dpp4: Yes | 1.27 | 0.81 | 1.98 | 0.11 | 0.11 |  |  |  |
| Egfr < 60 ml/min/1.73 m^2^ | 0.88 | 0.69 | 1.13 | 0.12 | 0.26 | 0.88 | 0.75 | 1.00 |
| Egfr in [60; 90) ml/min/1.73 m^2^ | 0.76 | 0.61 | 0.94 | 0.20 | 0.52 |  |  |  |
| Egfr ≥ 90 ml/min/1.73 m^2^ | 1.10 | 0.77 | 1.57 | 0.13 | 0.22 |  |  |  |
| Ethnicity: Not Hispanic/Latinos | 0.91 | 0.77 | 1.07 | 1.00 | 0.82 | 0.91 | 0.79 | 1.03 |
| Region: Europe | 1.02 | 0.81 | 1.28 | 1.00 | 0.41 | 1.02 | 0.90 | 1.14 |
| Hba1c < 8.5 % | 0.76 | 0.64 | 0.90 | 0.44 | 0.69 | 0.81 | 0.69 | 0.93 |
| Hba1c ≥ 8.5 % | 1.14 | 0.86 | 1.50 | 0.08 | 0.31 |  |  |  |
| Insulin: No | 0.79 | 0.64 | 0.97 | 0.31 | 0.52 | 0.83 | 0.71 | 0.95 |
| Insulin: Yes | 0.93 | 0.75 | 1.13 | 0.16 | 0.48 |  |  |  |
| Metformin: No | 0.72 | 0.56 | 0.94 | 0.13 | 0.26 | 0.86 | 0.73 | 0.98 |
| Metformin: Yes | 0.92 | 0.77 | 1.10 | 0.33 | 0.74 |  |  |  |
| MI or stroke: No | 0.88 | 0.66 | 1.18 | 0.91 | 0.35 | 0.88 | 0.75 | 1.01 |
| MI or stroke: Yes | 0.84 | 0.71 | 1.00 | 0.09 | 0.65 |  |  |  |
| peripheral artery disease | 0.94 | 0.47 | 1.88 | 0.06 | 0.09 | 0.94 | 0.77 | 1.11 |
| SBP ≥140 mmHg and/or  DBP ≥90 mmHg | 0.83 | 0.66 | 1.03 | 0.20 | 0.39 | 0.83 | 0.72 | 0.94 |
| SBP <140 mmHg and  DBP <90 mmHg | 0.89 | 0.73 | 1.08 | 0.20 | 0.61 | 0.89 | 0.76 | 1.02 |
| Sex: Male | 0.87 | 0.73 | 1.02 | 0.56 | 0.71 | 0.85 | 0.73 | 0.97 |
| Sex: Female | 0.83 | 0.62 | 1.11 | 0.44 | 0.29 |  |  |  |
| Statins: No | 0.79 | 0.59 | 1.07 | 0.16 | 0.23 | 0.84 | 072 | 0.97 |
| Statins: Yes | 0.88 | 0.74 | 1.04 | 0.26 | 0.77 |  |  |  |
| Sulfonylurea: No | 0.85 | 0.70 | 1.02 | 0.34 | 0.57 | 0.86 | 0.73 | 0.98 |
| sulfonylurea : Yes | 0.87 | 0.69 | 1.11 | 0.13 | 0.43 |  |  |  |
| Thiazolidinediones: No | 0.85 | 0.73 | 0.98 | 0.44 | 0.96 | 0.86 | 0.74 | 0.98 |
| Thiazolidinediones: Yes | 1.13 | 0.55 | 2.31 | 0.02 | 0.04 |  |  |  |
| Albumin creatinine ratio < 30 mg/g | 0.89 | 0.72 | 1.10 | 0.29 | 0.59 | 0.89 | 0.76 | 1.02 |
| Albumin creatinine ratio 30-300 mg/g | 0.89 | 0.69 | 1.16 | 0.16 | 0.29 |  |  |  |
| Albumin creatinine ratio >300 mg/g | 0.69 | 0.49 | 0.96 | 0.00 | 0.11 |  |  |  |
| Race: White | 0.88 | 0.74 | 1.04 | 1.00 | 0.72 | 0.88 | 0.76 | 1.00 |
|  | **CVOT estimate : 0.86 (0.74-0.99)** | | | | | **Transposed estimate: 0.88 (0.74-1.03)** | | |

**Table S6. LEADER**. CVOT cardiovascular outcome trial, CVD cardiovascular disease, BMI Body Mass Index, HR hazard ratio, low CI and high CI refer to the low and high 95% confidence interval (CI) limit, respectively.

| **Characteristic*s*** | **CVOT estimates** | | | **Proportions** | | **Sub-group weighted mean of strato-specific CVOT estimates** | | |
| --- | --- | --- | --- | --- | --- | --- | --- | --- |
|  | *HR* | *low CI* | *high CI* | *Darwin* | *CVOT* | *HR* | *low CI* | *high CI* |
| Sex: Female | 0.88 | 0.72 | 1.08 | 0.44 | 0.36 | 0.87 | 0.76 | 0.98 |
| Sex: Male | 0.86 | 0.75 | 0.98 | 0.56 | 0.64 |  |  |  |
| Age < 60 yrs | 0.78 | 0.62 | 0.97 | 0.20 | 0.25 | 0.87 | 0.77 | 0.98 |
| Age ≥ 60 yrs | 0.90 | 0.79 | 1.02 | 0.79 | 0.75 |  |  |  |
| BMI < 30 kg/m^2^ | 0.96 | 0.81 | 1.15 | 0.29 | 0.38 | 0.90 | 0.79 | 1.01 |
| BMI ≥ 30 kg/m^2^ | 0.82 | 0.71 | 0.94 | 0.20 | 0.62 |  |  |  |
| Hba1c ≤ 8.3 % | 0.89 | 0.76 | 1.05 | 0.43 | 0.51 | 0.88 | 0.77 | 0.99 |
| Hba1c > 8.3 % | 0.84 | 0.72 | 0.98 | 0.09 | 0.49 |  |  |  |
| Duration diabetes ≤ 11 yrs | 0.82 | 0.70 | 0.97 | 0.58 | 0.47 | 0.85 | 0.75 | 0.96 |
| Duration diabetes > 11 yrs | 0.90 | 0.78 | 1.04 | 0.42 | 0.52 |  |  |  |
| CVD: Yes | 0.83 | 0.74 | 0.93 | 0.20 | 0.81 | 0.83 | 0.73 | 0.93 |
| CVD Risk factors: Yes | 1.20 | 0.86 | 1.67 | 0.10 | 0.19 | 1.20 | 1.06 | 1.34 |
| Egfr < 60 ml/min/1.73 m^2^ | 0.69 | 0.57 | 0.85 | 0.12 | 0.23 | 0.86 | 0.76 | 0.98 |
| Egfr ≥ 60 ml/min/1.73 m^2^ | 0.94 | 0.83 | 1.07 | 0.33 | 0.77 |  |  |  |
| Heart Failure: No | 0.85 | 0.76 | 0.96 | 0.98 | 0.86 | 0.85 | 0.74 | 0.96 |
| Heart Failure: Yes | 0.94 | 0.72 | 1.21 | 0.02 | 0.14 |  |  |  |
| Antidiabetic therapy: 1 oral | 0.75 | 0.58 | 0.98 | 0.17 | 0.19 | 0.83 | 0.73 | 0.94 |
| Antidiabetic therapy: more than 1 oral | 0.95 | 0.78 | 1.16 | 0.14 | 0.32 |  |  |  |
| Antidiabetic therapy: Insulin with oral | 0.89 | 0.74 | 1.06 | 0.09 | 0.37 |  |  |  |
| Antidiabetic therapy: Insulin without oral | 0.86 | 0.63 | 1.17 | 0.07 | 0.08 |  |  |  |
| Antidiabetic therapy: None | 0.73 | 0.42 | 1.25 | 0.04 | 0.04 |  |  |  |
| Region: Europe | 0.82 | 0.68 | 0.98 | 1.00 | 0.35 | 0.82 | 0.72 | 0.92 |
| Race: White | 0.90 | 0.80 | 1.02 | 1.00 | 0.77 | 0.90 | 0.79 | 1.01 |
| Ethnicity: Non-Hispanic | 0.89 | 0.79 | 1.00 | 1.00 | 0.88 | 0.89 | 0.78 | 1.00 |
|  | **CVOT estimate : 0.87 (0.78-0.97)** | | | | | **Transposed estimate: 0.88 (0.77-0.99)** | | |

**Table S7. PIONEER-6**. CVOT cardiovascular outcome trial, CVD cardiovascular disease, BMI Body Mass Index, MI myocardial infarction, HR hazard ratio, low CI and high CI refer to the low and high 95% confidence interval (CI) limit, respectively.

| **Characteristic*s*** | **CVOT estimates** | | | **Proportions** | | **Sub-group weighted mean of strato-specific CVOT estimates** | | |
| --- | --- | --- | --- | --- | --- | --- | --- | --- |
|  | *HR* | *low CI* | *high CI* | *Darwin* | *CVOT* | *HR* | *low CI* | *high CI* |
| Age < 65 yrs | 0.51 | 0.29 | 0.90 | 0.26 | 0.42 | 0.85 | 0.66 | 1.05 |
| Age ≥ 65 yrs | 1.04 | 0.68 | 1.59 | 0.67 | 0.58 |  |  |  |
| Egfr < 60 ml/min/1.73 m^2^ | 0.74 | 0.41 | 1.33 | 0.12 | 0.27 | 0.79 | 0.60 | 0.98 |
| Egfr ≥ 60 ml/min/1.73 m^2^ | 0.81 | 0.54 | 1.22 | 0.33 | 0.73 |  |  |  |
| Sex: Female | 1.16 | 0.54 | 2.51 | 0.44 | 0.32 | 0.89 | 0.69 | 1.09 |
| Sex: Male | 0.72 | 0.50 | 1.05 | 0.56 | 0.68 |  |  |  |
| CVD: Yes | 0.83 | 0.58 | 1.17 | 0.20 | 0.85 | 0.83 | 0.65 | 1.01 |
| CVD risk factors: Yes | 0.51 | 0.15 | 1.68 | 0.10 | 0.15 | 0.51 | 0.25 | 0.77 |
| Hba1c < 8.6 % | 0.81 | 0.53 | 1.24 | 0.45 | 0.67 | 0.80 | 0.61 | 0.99 |
| Hba1c ≥ 8.6 % | 0.73 | 0.42 | 1.26 | 0.07 | 0.32 |  |  |  |
| BMI < 31 kg/m^2^ | 0.61 | 0.36 | 1.03 | 0.32 | 0.40 | 0.71 | 0.52 | 0.90 |
| BMI ≥ 31 kg/m^2^ | 0.95 | 0.61 | 1.48 | 0.16 | 0.60 |  |  |  |
| Race: White | 0.83 | 0.56 | 1.23 | 1.00 | 0.72 | 0.83 | 0.64 | 1.02 |
| MI or stroke: Yes | 0.97 | 0.64 | 1.49 | 0.09 | 0.45 | 0.62 | 0.41 | 0.82 |
| MI or stroke: No | 0.59 | 0.34 | 1.03 | 0.91 | 0.54 |  |  |  |
|  | **CVOT estimate : 0.79 (0.57-1.11)** | | | | | **Transposed estimate: 0.76 (0.41-1.10)** | | |

**Table S8. TECOS**. CVOT cardiovascular outcome trial, CVD cardiovascular disease, BMI Body Mass Index, MI myocardial infarction, SBP Systolic Blood Pressure, DBP Diastolic Blood Pressure, HR hazard ratio, low CI and high CI refer to the low and high 95% confidence interval (CI) limit, respectively.

| **Characteristic*s*** | **CVOT estimates** | | | **Proportions** | | **Sub-group weighted mean of strato-specific CVOT estimates** | | |
| --- | --- | --- | --- | --- | --- | --- | --- | --- |
|  | *HR* | *low CI* | *high CI* | *Darwin* | *CVOT* | *HR* | *low CI* | *high CI* |
| Age < 65 yrs | 0.95 | 0.82 | 1.11 | 0.32 | 0.45 | 0.99 | 0.87 | 1.11 |
| Age ≥ 65 yrs | 1.01 | 0.90 | 1.15 | 0.67 | 0.53 |  |  |  |
| Sex: Male | 0.99 | 0.88 | 1.10 | 0.56 | 0.71 | 0.97 | 0.86 | 1.09 |
| Sex: Female | 0.95 | 0.78 | 1.15 | 0.44 | 0.29 |  |  |  |
| Race: White | 0.97 | 0.87 | 1.08 | 1.00 | 0.68 | 0.97 | 0.86 | 1.08 |
| Region: Europe | 0.95 | 0.73 | 1.23 | 1.00 | 0.14 | 0.95 | 0.83 | 1.07 |
| Duration diabetes < 5 yrs | 0.99 | 0.78 | 1.26 | 0.27 | 0.19 | 0.99 | 0.87 | 1.11 |
| Duration diabetes is [5; 15) | 0.89 | 0.78 | 1.02 | 0.40 | 0.51 |  |  |  |
| Duration diabetes ≥ 15 yrs | 1.12 | 0.95 | 1.32 | 0.32 | 0.29 |  |  |  |
| Sulfonylurea: Yes | 0.99 | 0.86 | 1.14 | 0.13 | 0.45 | 0.98 | 0.86 | 1.09 |
| Sulfonylurea: No | 0.97 | 0.85 | 1.10 | 0.34 | 0.55 |  |  |  |
| Metformin: Yes | 0.96 | 0.83 | 1.04 | 0.33 | 0.82 | 1.01 | 0.91 | 1.10 |
| Metformin: No | 1.13 | 0.93 | 1.38 | 0.13 | 0.18 |  |  |  |
| Thiazolidinedione: Yes | 0.86 | 0.49 | 1.49 | 0.02 | 0.03 | 0.97 | 0.86 | 1.09 |
| Thiazolidinedione: No | 0.98 | 0.89 | 1.08 | 0.44 | 0.97 |  |  |  |
| Insulin: Yes | 1.01 | 0.85 | 1.21 | 0.16 | 0.23 | 0.98 | 0.86 | 1.10 |
| Insulin: No | 0.96 | 0.89 | 1.08 | 0.31 | 0.77 |  |  |  |
| Heart Failure: Yes | 0.97 | 0.80 | 1.17 | 0.02 | 0.18 | 0.99 | 0.88 | 1.10 |
| Heart Failure: No | 0.99 | 0.88 | 1.10 | 0.98 | 0.82 |  |  |  |
| Hba1c < 7.2 % | 0.95 | 0.83 | 1.09 | 0.29 | 0.52 | 0.97 | 0.86 | 1.09 |
| Hba1c ≥ 7.2 % | 1.00 | 0.88 | 1.14 | 0.23 | 0.48 |  |  |  |
| Egfr < 60 ml/min/1.73 m^2^ | 0.92 | 0.78 | 1.10 | 0.12 | 0.23 | 0.98 | 0.86 | 1.10 |
| Egfr ≥ 60 ml/min/1.73 m^2^ | 1.00 | 0.89 | 1.12 | 0.33 | 0.76 |  |  |  |
| SBP < 140 mmHg | 0.96 | 0.85 | 1.09 | 0.20 | 0.60 | 0.98 | 0.86 | 1.10 |
| SBP in [140; 160) mmHg | 1.03 | 0.87 | 1.23 | 0.13 | 0.31 |  |  |  |
| SBP ≥ 160 mmHg | 0.92 | 0.70 | 1.23 | 0.06 | 0.09 |  |  |  |
| DBP <90 mmHg | 0.98 | 0.88 | 1.09 | 0.34 | 0.85 | 0.97 | 0.85 | 1.09 |
| DBP in [90; 100) mmHg | 1.08 | 0.84 | 1.40 | 0.04 | 0.13 |  |  |  |
| DBP ≥ 100 mmHg | 0.51 | 0.25 | 1.02 | 0.01 | 0.02 |  |  |  |
| BMI < 30 kg/m^2^ | 1.08 | 0.95 | 1.24 | 0.29 | 0.53 | 0.99 | 0.88 | 1.11 |
| BMI ≥ 30 | 0.88 | 0.76 | 1.01 | 0.20 | 0.46 |  |  |  |
| Statins: Yes | 0.98 | 0.88 | 1.10 | 0.26 | 0.80 | 0.97 | 0.85 | 1.09 |
| Statins: No | 0.96 | 0.79 | 1.16 | 0.16 | 0.20 |  |  |  |
| ACE inhibitors: Yes | 1.00 | 0.90 | 1.11 | 0.27 | 0.79 | 0.96 | 0.85 | 1.08 |
| ACE inhibitors: No | 0.89 | 0.71 | 1.11 | 0.14 | 0.21 |  |  |  |
| Diuretics: Yes | 0.96 | 0.84 | 1.09 | 0.08 | 0.41 | 1.00 | 0.89 | 1.11 |
| Diuretics: No | 1.01 | 0.88 | 1.15 | 0.34 | 0.59 |  |  |  |
| Calcium channel blockers: Yes | 0.93 | 0.79 | 1.09 | 0.10 | 0.34 | 0.99 | 0.88 | 1.10 |
| Calcium channel blockers: No | 1.01 | 0.89 | 1.13 | 0.31 | 0.66 |  |  |  |
| Beta blockers: Yes | 0.96 | 0.85 | 1.07 | 0.13 | 0.64 | 1.01 | 0.90 | 1.13 |
| Beta blockers: No | 1.04 | 0.87 | 1.23 | 0.29 | 0.36 |  |  |  |
|  | **CVOT estimate: 0.98 (0.88-1.09)** | | | | | **Transposed estimate 0.97 (0.87-1.06)** | | |

**Table S9. SAVOR-TIMI**. CVOT cardiovascular outcome trial, BMI Body Mass Index, HR hazard ratio, Low CI and high CI refer to the low and high 95% confidence interval (CI) limit, respectively.

| **Characteristic*s*** | **CVOT estimates** | | | **Proportions** | | **Sub-group weighted mean of strato-specific CVOT estimates** | | |
| --- | --- | --- | --- | --- | --- | --- | --- | --- |
|  | *HR* | *low CI* | *high CI* | *Darwin* | *CVOT* | *HR* | *low CI* | *high CI* |
| Egfr < 30 ml/min/1.73 m^2^ | 0.83 | 0.49 | 1.39 | 0.02 | 0.02 | 1.00 | 0.85 | 1.15 |
| Egfr in [30;50) ml/min/1.73 m^2^ | 1.02 | 0.79 | 1.30 | 0.05 | 0.14 |  |  |  |
| Egfr ≥ 50 ml/min/1.73 m^2^ | 1.01 | 0.88 | 1.15 | 0.37 | 0.84 |  |  |  |
| Sex: Male | 1.01 | 0.89 | 1.16 | 0.56 | 0.67 | 0.99 | 0.84 | 1.14 |
| Sex: Female | 0.97 | 0.78 | 1.20 | 0.44 | 0.33 |  |  |  |
| Race: White | 0.98 | 0.86 | 1.11 | 1.00 | 0.75 | 0.98 | 0.84 | 1.12 |
| Age < 75 yrs | 1.01 | 0.89 | 1.15 | 0.63 | 0.86 | 0.99 | 0.84 | 1.14 |
| Age ≥ 75 yrs | 0.96 | 0.75 | 1.22 | 0.36 | 0.14 |  |  |  |
| Region: Europe | 0.96 | 0.81 | 1.13 | 1.00 | 0.42 | 0.96 | 0.82 | 1.10 |
| BMI < 30 kg/m^2^ | 1.01 | 0.86 | 1.19 | 0.28 | 0.46 | 1.00 | 0.85 | 1.15 |
| BMI ≥ 30 kg/m^2^ | 0.99 | 0.85 | 1.16 | 0.20 | 0.53 |  |  |  |
| Heart Failure: Yes | 1.13 | 0.89 | 1.43 | 0.02 | 0.13 | 0.97 | 0.83 | 1.12 |
| 2Heart Failure: No | 0.97 | 0.85 | 1.10 | 0.98 | 0.87 |  |  |  |
| Duration diabetes < 5 yrs | 1.07 | 0.82 | 1.40 | 0.27 | 0.24 | 1.01 | 0.86 | 1.16 |
| Duration diabetes in [5; 10) yrs | 1.04 | 0.81 | 1.33 | 0.22 | 0.24 |  |  |  |
| Duration diabetes in [10;15) yrs | 0.94 | 0.74 | 1.19 | 0.18 | 0.21 |  |  |  |
| Duration diabetes in [15;20) yrs | 1.06 | 0.79 | 1.41 | 0.12 | 0.13 |  |  |  |
| Duration diabetes ≥20 yrs | 0.93 | 0.74 | 1.17 | 0.20 | 0.18 |  |  |  |
| Hba1c < 7 % | 1.01 | 0.78 | 1.31 | 0.24 | 0.25 | 1.01 | 0.86 | 1.15 |
| Hba1c in[7; 8) % | 0.98 | 0.80 | 1.20 | 0.16 | 0.33 |  |  |  |
| Hba1c in [8; 9) % | 1.09 | 0.85 | 1.39 | 0.07 | 0.19 |  |  |  |
| Hba1c ≥ 9 % | 0.95 | 0.77 | 1.18 | 0.05 | 0.21 |  |  |  |
| Insulin: Yes | 1.03 | 0.88 | 1.20 | 0.16 | 0.41 | 0.98 | 0.84 | 1.13 |
| Insulin: No | 0.96 | 0.82 | 1.13 | 0.31 | 0.59 |  |  |  |
| Sulfonylurea: Yes | 0.95 | 0.79 | 1.14 | 0.13 | 0.40 | 1.01 | 0.86 | 1.15 |
| Sulfonylurea: No | 1.03 | 0.90 | 1.19 | 0.34 | 0.60 |  |  |  |
| Metformin: Yes | 0.97 | 0.84 | 1.13 | 0.33 | 0.70 | 0.99 | 0.84 | 1.15 |
| Metformin: No | 1.05 | 0.88 | 1.25 | 0.13 | 0.30 |  |  |  |
| Thiazolidinedione: Yes | 0.59 | 0.33 | 1.04 | 0.02 | 0.06 | 0.99 | 0.84 | 1.14 |
| Thiazolidinedione: No | 1.02 | 0.91 | 1.15 | 0.44 | 0.94 |  |  |  |
| Micro-albumin creatinine ratio < 30 mg/g | 1.07 | 0.90 | 1.27 | 0.29 | 0.59 | 1.01 | 0.85 | 1.16 |
| Micro-albumin creatinine ratio in [30; 300) mg/g | 0.90 | 0.74 | 1.09 | 0.16 | 0.27 |  |  |  |
| Micro-albumin creatinine ratio ≥ 300 mg/g | 0.88 | 0.68 | 1.13 | 0.00 | 0.10 |  |  |  |
| Ethnicity: Not hispanic | 0.97 | 0.86 | 1.10 | 1.00 | 0.79 | 0.97 | 0.83 | 1.11 |
| Weight < 80 Kg | 1.10 | 0.91 | 1.33 | 0.25 | 0.36 | 1.05 | 0.88 | 1.17 |
| Weight ≥ 80 Kg | 0.95 | 0.83 | 1.09 | 0.24 | 0.64 |  |  |  |
| Hypertension: Yes | 0.97 | 0.86 | 1.10 | 0.34 | 0.82 | 1.00 | 0.85 | 1.15 |
| Hypertension: No | 1.14 | 0.87 | 1.51 | 0.08 | 0.18 |  |  |  |
| Statins: Yes | 0.99 | 0.87 | 1.12 | 0.26 | 0.78 | 1.01 | 0.87 | 1.15 |
| Statins: No | 1.04 | 0.80 | 1.34 | 0.16 | 0.22 |  |  |  |
| ACEi/ARB: Yes | 0.98 | 0.86 | 1.11 | 0.27 | 0.79 | 1.01 | 0.87 | 1.16 |
| ACEi/ARB: No | 1.08 | 0.85 | 1.38 | 0.14 | 0.21 |  |  |  |
| Diuretics: Yes | 1.02 | 0.88 | 1.18 | 0.08 | 0.44 | 0.99 | 0.84 | 1.14 |
| Diuretics: No | 0.98 | 0.82 | 1.16 | 0.34 | 0.56 |  | | |
|  | **CVOT estimate : 1.00 (0.89-1.12)** | | | | | **Transposed estimate: 0.99 (0.87-1.10)** | | |

**Table S10. EXCEL**. CVOT cardiovascular outcome trial, CVD cardiovascular disease, BMI Body Mass Index, HR hazard ratio, low CI and high CI refer to the low and high 95% confidence interval (CI) limit, respectively.

| **1Characteristic*s*** | **CVOT estimates** | | | **Proportions** | | **Sub-group weighted mean of strato-specific CVOT estimates** | | |
| --- | --- | --- | --- | --- | --- | --- | --- | --- |
|  | *HR* | *low CI* | *high CI* | *Darwin* | *CVOT* | *HR* | *low CI* | *high CI* |
| Age < 65 yrs | 1.05 | 0.92 | 1.21 | 0.32 | 0.60 | 0.87 | 0.75 | 0.99 |
| Age ≥ 65 yrs | 0.80 | 0.71 | 0.91 | 0.67 | 0.40 |  |  |  |
| Sex: Male | 0.94 | 0.84 | 1.05 | 0.56 | 0.62 | 0.90 | 0.79 | 1.02 |
| Sex: Female | 0.86 | 0.73 | 1.03 | 0.44 | 0.38 |  |  |  |
| Race: White | 0.95 | 0.85 | 1.05 | 1.00 | 0.76 | 0.95 | 0.84 | 1.06 |
| Region: Europe | 1.00 | 0.87 | 1.15 | 1.00 | 0.46 | 1.00 | 0.88 | 1.12 |
| Duration diabetes < 5 yrs | 0.70 | 0.50 | 0.97 | 0.27 | 0.14 | 0.87 | 0.75 | 0.99 |
| Duration diabetes in [5;15) yrs | 0.98 | 0.85 | 1.12 | 0.40 | 0.49 |  |  |  |
| Duration diabetes ≥ 15 yrs | 0.90 | 0.79 | 1.04 | 0.32 | 0.37 |  |  |  |
| Anti-hyperglycemic oral agent therapy: Yes | 0.93 | 0.84 | 1.04 | 0.39 | 0.85 | 0.85 | 0.75 | 1.00 |
| Anti-hyperglycemic oral agent therapy: No | 0.84 | 0.69 | 1.03 | 0.61 | 0.15 |  |  |  |
| Insulin: Yes | 0.89 | 0.78 | 1.00 | 0.16 | 0.46 | 0.93 | 0.81 | 1.05 |
| Insulin: No | 0.95 | 0.83 | 1.10 | 0.31 | 0.54 |  |  |  |
| Dpp4: Yes | 1.08 | 0.84 | 1.39 | 0.11 | 0.15 | 0.93 | 0.81 | 1.05 |
| Dpp4: No | 0.89 | 0.80 | 0.99 | 0.36 | 0.85 |  |  |  |
| Heart Failure: Yes | 0.97 | 0.81 | 1.16 | 0.02 | 0.16 | 0.90 | 0.79 | 1.01 |
| Heart Failure: No | 0.90 | 0.81 | 1.00 | 0.98 | 0.84 |  |  |  |
| Hba1c < 8 % | 0.91 | 0.80 | 1.05 | 0.40 | 0.49 | 0.91 | 0.79 | 1.03 |
| Hba1c ≥ 8% | 0.91 | 0.80 | 1.04 | 0.12 | 0.51 |  |  |  |
| Egfr < 60 ml/min/1.73 m^2^ | 1.01 | 0.86 | 1.19 | 0.12 | 0.22 | 0.90 | 0.77 | 1.02 |
| Egfr ≥ 60 ml/min/1.73 m^2^ | 0.86 | 0.77 | 0.97 | 0.33 | 0.78 |  |  |  |
| BMI < 30 kg/m^2^ | 0.94 | 0.79 | 1.10 | 0.28 | 0.36 | 0.92 | 0.81 | 1.03 |
| BMI ≥ 30 kg/m^2^ | 0.89 | 0.79 | 1.00 | 0.20 | 0.63 |  |  |  |
| CVD: Yes | 0.90 | 0.82 | 1.00 | 0.20 | 0.73 | 0.97 | 0.84 | 1.10 |
| CVD: No | 0.99 | 0.77 | 1.28 | 0.80 | 0.27 |  |  |  |
|  | **CVOT estimate: 0.92 (0.82-1.02)** | | | | | **Transposed estimate: 0.91 (0.83-1.00)** | | |
